# Chemical Colitis Induced by Low-dose Hydrogen Peroxide Enema in a Cocaine User

**DOI:** 10.7759/cureus.7017

**Published:** 2020-02-17

**Authors:** Jason Galo, Michelle Zaydlin, Diego A Celli-Cabada

**Affiliations:** 1 Internal Medicine, University of Miami Miller School of Medicine/Jackson Memorial Hospital, Miami, USA; 2 Psychiatry, University of Miami Miller School of Medicine/Jackson Memorial Hospital, Miami, USA; 3 Internal Medicine, University of Miami Miller School of Medicine, Miami, USA

**Keywords:** chemical colitis, hydrogen peroxide, enema, cocaine abuse, healthcare inequality, hematochezia

## Abstract

Home remedies are usually cheap options to alleviate conditions commonly used prior to patients seeking medical advice for their problems, and are sometimes the only option for some populations that have otherwise no healthcare access. Hydrogen peroxide enemas appear to be an easily accessible solution to constipation, with “how to guides” found ubiquitously on the Internet. To our knowledge there are a few case reports exposing its complications. Our case reports complications in a patient who used a lower than average dose of a hydrogen peroxide enema, albeit compounded by cocaine abuse. Our experience suggests that the risks of concurrent use of cocaine and hydrogen peroxide enemas can lead to dangerous vasoconstriction, decreased blood flood to the bowel mucosa, and might lead to significant complications to otherwise tolerable doses of corrosive agents.

## Introduction

Constipation is a common complaint in the general population presented to medical professionals. Easy to find, non-FDA-approved Internet solutions and home remedies are frequently used as a quick and cheap option. Often times these remedies are used by patients with limited healthcare access which may impede adequate treatment, and this can sometimes lead to fatal complications [1].

Cocaine abuse is a major healthcare problem that can lead to cardiovascular complications such as myocardial infarction, arrhythmias and stroke. Cocaine also has a systemic vasoconstrictive effect that disrupts almost every system’s physiology, inducing susceptibility to lower than normal insults [2].

Hydrogen peroxide enemas have been associated with chemical colitis, especially if performed on a friable mucosa after cocaine abuse, and this can lead to catastrophic complications. Patient education is of utmost importance to provide a safe and effective control of symptoms while avoiding preventable complications [3,4].

## Case presentation

A 60-year-old African American female with past medical history of cocaine use disorder and self-treated chronic constipation by bi-weekly to monthly satisfactory and uncomplicated use of hydrogen peroxide enemas, presented to the emergency department with a one-day narrative of left-sided abdominal pain and bright red blood per rectum.

The patient reported that symptoms began the morning prior to admission. At that time, she experienced bloating and constipation, and she purchased over-the-counter 3% hydrogen peroxide at the local pharmacy which she mixed in a 1:5 fashion with water and used a dish-soap bottle to irrigate the solution into her rectum. She reported frequently having done this in the past to alleviate symptoms of constipation and bloating. Shortly after the administration of the enema, the patient began to expel half a cup of bright red blood per rectum mixed with clots. She had a total of six episodes before deciding to seek medical attention.

**Figure 1:**
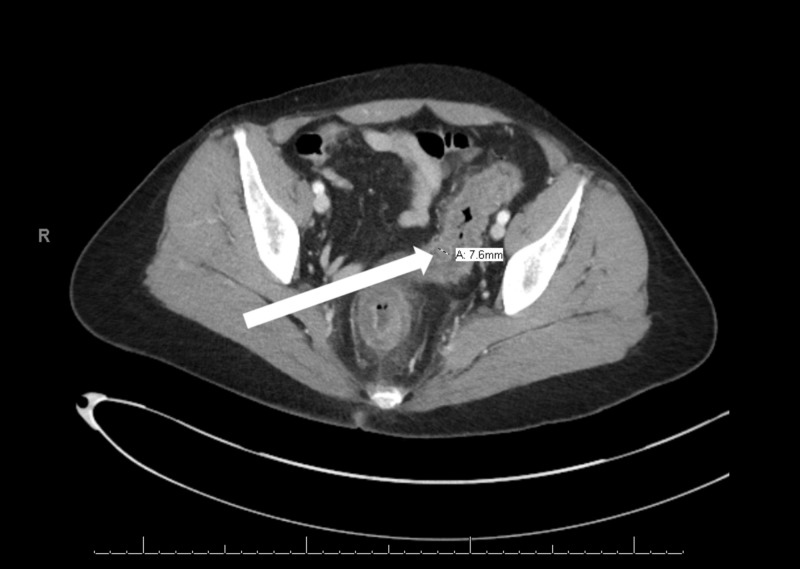
CT abdomen and pelvis with contrast scan showing the anal canal and entire sigmoid colon with circumferential wall thickening measuring up to 7 mm (arrow) with minimal adjacent fat stranding without focal collection of free air.

Upon interview, she endorsed a relapse with cocaine use just prior to symptom onset. Physical examination showed tachycardia and hypotension with left lower quadrant abdominal tenderness with guarding but no rebound. Basic metabolic panel was significant for hypokalemia and hypochloremia with positive urine toxicology for cocaine and a normal lactic acid. A computerized tomography of abdomen and pelvis with contrast revealed moderate inflammation and wall thickening of the anal canal and sigmoid colon (Figure 1).

The patient was admitted and gastroenterology was consulted, recommending intravenous fluid hydration, intravenous proton pump inhibitors and clear liquid diet without any further emergency interventions. The patient achieved symptomatic relief over the course of two days. She was discharged with formal education on constipation management and outpatient follow-up.

## Discussion

Cocaine is a highly addictive drug classified as a Schedule II medication under the Controlled Substances Act in the United States, and has been reported to be used by 0.4% of the world population [5]. It enhances the monoamine neurotransmitters in central and peripheral receptors inducing potent vasoconstriction [6]. Cocaine-induced vasoconstriction and ischemia may result in dose-dependent gastrointestinal ulceration, infarction, perforation, and ischemic colitis, and friable mucosa is more susceptible to transient insults [7, 8].

Constipation is a common complaint among adult individuals; health disparities, inaccessible health care and lack of education are some factors that predispose patients to look for alternative sources such as the Internet to ameliorate their complaints. Home remedies are particularly popular in groups that have often experienced barriers to receiving health care or who have experienced discrimination by the health care system [9]. These groups include, but are not limited to, older adults, African Americans and patients with substance use disorders. Home remedies are often easily found via the Internet and once patients experience relief, they tend to continue using the same remedy. This can perpetuate a cycle of avoiding to seek professional medical assistance. Self-administered home remedies such as hydrogen peroxide enemas have been reported as a cause of chemical colitis, which was seen in the case described above. In recent literature reviews, several cases of both adult and pediatric have been described, with the classic presentation entailing a triad of abdominal pain, bloody diarrhea, and tenesmus [3,4]. In the best-case scenario, symptoms are reported to improve with this homeopathic management. However, using hydrogen peroxide enemas, especially concomitantly with cocaine, can cause severe colitis, enough to cause bowel perforation.

In the present case, our patient used a lower than web-recommended and previously tolerated dose; commonly between 1:1 to 1:3 dilution, patient’s dose was 1:5 [3]. Given her frequent and chronic use of hydrogen peroxide enema without complications, it is suspected that her concurrent use of cocaine likely contributed to friability of the mucosa leading to the clinical picture and aforementioned symptoms.

## Conclusions

This case demonstrates an unusual presentation of chemical-colitis that was likely secondary to both acute on chronic hydrogen peroxide enemas on a cocaine abuser where medical management alone led to resolution of symptoms in a relatively short time period. Education on constipation management is of utmost importance. In particular, patient education on the potential risks of home remedies that can lead to severe complications is warranted.
